# Circulating miRNAs as Diagnostic Biomarkers for Parkinson’s Disease

**DOI:** 10.3389/fnins.2018.00625

**Published:** 2018-09-05

**Authors:** Anna Elisa Roser, Lucas Caldi Gomes, Jonas Schünemann, Fabian Maass, Paul Lingor

**Affiliations:** ^1^Department of Neurology, University Medical Center Göttingen, Göttingen, Germany; ^2^DFG Cluster of Excellence Nanoscale Microscopy and Molecular Physiology of the Brain, University Medical Center Göttingen, Göttingen, Germany; ^3^German Center for Neurodegenerative Diseases, Göttingen, Germany

**Keywords:** biomarker, miRNA, Parkinson’s disease, Parkinsonian syndromes, neurodegeneration, blood, CSF

## Abstract

Parkinson’s disease (PD) is the second most common neurodegenerative disorder worldwide. Its main neuropathological hallmarks are the degeneration of dopaminergic neurons in the substantia nigra and alpha-synuclein containing protein inclusions, called Lewy Bodies. The diagnosis of idiopathic PD is still based on the assessment of clinical criteria, leading to an insufficient diagnostic accuracy. Additionally, there is no biomarker available allowing the prediction of the disease course or monitoring the response to therapeutic approaches. So far, protein biomarker candidates such as alpha-synuclein have failed to improve diagnosis of PD. Circulating microRNAs (miRNAs) in body fluids are promising biomarker candidates for PD, as they are easily accessible by non- or minimally-invasive procedures and changes in their expression are associated with pathophysiological processes relevant for PD. Advances in miRNA analysis methods resulted in numerous recent publications on miRNAs as putative biomarkers. Here, we discuss the applicability of different body fluids as sources for miRNA biomarkers, highlight technical aspects of miRNA analysis and give an overview on published studies investigating circulating miRNAs as biomarker candidates for diagnosis of PD and other Parkinsonian syndromes.

## Introduction

Parkinson’s disease (PD) is a progressive neurodegenerative disorder and affects about 0.3% of the population in industrialized countries (Dexter and Jenner, 2013). Due to the lack of a reliable objective biomarker, the diagnosis of idiopathic PD is still based on the assessment of clinical criteria (Postuma et al., 2015), leading to an insufficient diagnostic accuracy especially in the early stages of the disease (Rizzo et al., 2016). Furthermore, no biochemical marker currently is able to predict the disease course, the individual response to therapy or contributes to a clear differentiation between idiopathic PD and clinical mimics, such as atypical Parkinsonian syndromes. The neuropathology of PD is characterized by a progressive loss of dopaminergic neurons in the substantia nigra and their striatal projections, as well as intra-neuronal α-synuclein-containing protein inclusions, called Lewy Bodies. To establish a clinically useful biomarker for PD, the use of body fluids that are available by non- or minimally-invasive procedures (e.g., cerebrospinal fluid (CSF), blood, saliva or urine) would be preferable. One of the most extensively tested candidates as a liquid biomarker related to PD pathology is α-synuclein. However, several studies showed that α-synuclein levels in blood or CSF lack discrimination power and specificity (Malek et al., 2014; Goldman et al., 2018). Next to proteins and metabolites, circulating microRNAs (miRNAs) were previously shown to be useful biomarkers in other pathologies, e.g., cancer (Jamali et al., 2018) or cardiovascular disease (de Gonzalo-Calvo et al., 2018). miRNAs are small non-coding RNAs involved in the regulation of gene expression. Changes in the expression of miRNAs are associated with PD-relevant pathophysiological processes, thus they are auspicious body fluid-derived biomarkers for diagnosis and progression of PD. In this review, we discuss the suitability of different body fluids as a source for miRNA biomarkers, highlight technical aspects of RNA isolation, detection and analyses and give an overview of recent advances in miRNA-biomarker research relevant for PD.

## Sources, Technical Considerations and Biomarker Potential of mIRNAs

miRNAs are endogenous post-transcriptional regulators of gene expression that are crucial for biological processes. The expression of different miRNAs is strongly dependent on physiological and pathological stimuli and reflects the functional state of a cell, making the miRNA signature an interesting biomarker candidate. Because the affected CNS tissue itself is not routinely accessible it is important to consider that any body fluid that is used as biomarker source only partially recapitulates CNS pathology. Possible sources for miRNAs include non-neuronal cells or easily accessible body fluids. One of the best studied non-neuronal cell types in neurodegenerative disease biomarker research are peripheral blood mononuclear cells (PBMCs), which contain lymphocytes and monocytes. Several studies investigated the comparability of genetic and epigenetic signatures in the CNS and blood, at which epigenomic changes like DNA methylation showed a higher correlation than transcriptomic changes [reviewed in (Tylee et al., 2013)]. Furthermore it was shown that analysis of PBMC miRNA expression allows to discriminate controls from diseased patients in different neurological disorders (Keller et al., 2009; Gandhi et al., 2013; Fan et al., 2014, 2015; Lai et al., 2016; Ren et al., 2016). Consequently, miRNA expression in PBMCs was suggested as a diagnostic biomarker for PD.

The recent progress in high-sensitive RNA detection and analyses technologies led to the discovery of extracellular RNA species in body fluids, among them messenger RNA (mRNA), ribosomal RNA (rRNA), long non-coding RNA (lncRNA), exonic circular RNA (circRNA) and small non-coding RNA species like miRNA reviewed in Patton et al. (2015). The majority of extracellular RNA species are detected in vesicles such as exosomes, nano- and microvesicles or apoptotic bodies; however, they can also be found outside of vesicular structures bound to lipoproteins (Vickers et al., 2011) or Argonaute2 (Arroyo et al., 2011). Several studies reported the isolation of extracellular miRNA from different body fluids such as saliva, serum, urine, and CSF (Burgos et al., 2014; Akers et al., 2017; Dangla-Valls et al., 2017; Yeri et al., 2017). Because CSF circulates in a closed system and is in direct exchange with the brain parenchyma, it is a promising biomarker source for neurodegenerative diseases that might provide more specific insights into the cellular disease-mechanisms as compared to blood. miRNA profiles in CSF were shown to discriminate healthy controls from patients with different neurological diseases (Dangla-Valls et al., 2017; Marques et al., 2017; Reed et al., 2018).

Next to the choice of the biomarker source, another important aspect is the technique for RNA isolation and analysis. Using extracellular miRNAs means to either isolate them directly from the source material like CSF or isolate vesicles prior to RNA isolation to achieve an enrichment. Different methods are available to isolate vesicles from body fluids, among them differential ultracentrifugation, density gradient-based ultracentrifugation or precipitation-based methods. A comparison of the resulting vesicular RNA profiles showed marked differences depending on the vesicular isolation technique (Van Deun et al., 2014), which is likely due to different RNA profiles in respective vesicle subpopulations (Crescitelli et al., 2013). Additionally, the RNA isolation method plays an important role. Most methods rely on either guanidinium thiocyanate-phenol-chloroform extraction or a lysis step followed by column precipitation and result in high quality RNA. However, they also lead to differences in terms of RNA quantity and size profiles, due to, e.g., lysis capacities of buffers (Eldh et al., 2012; Burgos et al., 2013; McAlexander et al., 2013).

**FIGURE 1:**
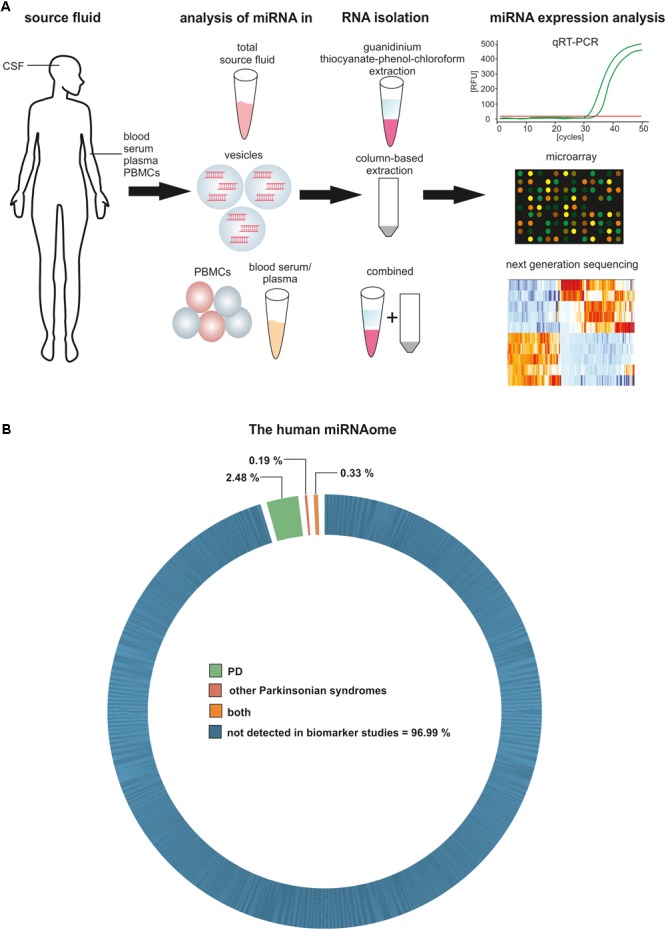
Circulating miRNAs as biomarkers for PD and other Parkinsonian syndromes. **(A)** Methodological aspects to consider in circulating biomarker research. **(B)** Proportion of the human miRNAome demonstrated to be significantly regulated in biomarker studies for PD and other Parkinsonian syndromes.

The precise determination of miRNA expression levels in a specimen is essential for the development of miRNA-based biomarkers. Several miRNA detection methods have been developed, from solid-based methods like Northern blots and microarrays, to solution-based methods like PCR and next generation sequencing (NGS), and all of them come with different limitations reviewed in de Planell-Saguer and Rodicio (2011). Ideally, the method of choice is highly sensitive and allows quantitative and qualitative unbiased analysis of miRNAs, even with minimal amounts of input material. To date, the most promising method used is small RNA sequencing, because it allows the unbiased analysis of all known and unknown miRNAs in a specimen without target pre-selection. Shortcomings for biomarker research are the RNA input requirements for sequencing protocols as well as the still relatively high costs of NGS. Some studies demonstrate that downscaling is possible without losing sensitivity and specificity (Burgos et al., 2013, 2014; Rao et al., 2013; dos Santos et al., 2018). As NGS is still a relatively new technique and the solutions for small RNA sequencing are continuously improved, it is likely that this technique will be more widely available in the future. The development of diagnostic biomarkers for PD based on circulating miRNAs thus requires the consideration of multiple aspects like source liquid, isolation technique and quantification methods and these points should also be taken into account when comparing already published studies in this field (**Figure 1A**).

## Circulating miRNAs as Biomarkers in Parkinson’s Disease

So far, a limited number of studies have been published on miRNA expression in biological fluids from PD patients and only a small fraction of the human miRNAome has been detected in this context (**Figure 1B**). Blood and its derivatives were the most extensively studied source fluid. Only one study analyzed miRNA levels in whole blood by PCR arrays and revealed a set of differentially expressed miRNAs that permitted the discrimination of PD patients and controls (miR-1-3p, miR-22-5p, and miR-29a-3p), as well as differentiating between Levodopa/Carbidopa-treated and untreated PD groups (miR-16-2-3p, miR-26a-2-3p, and miR30a-5p) (Margis et al., 2011). Unfortunately, comparable studies validating these results were not published.

miRNA levels in plasma of PD patients were analyzed in four studies using either microarray or quantitative real-time PCR (qRT-PCR) for analysis. Interestingly, each of these studies reported different results without any overlap (**Supplementary Table 1**). The earliest study identified a set of PD-predictive miRNAs by microarrays (miR-1826, miR-626, and miR-505-3p) (Khoo et al., 2012). The candidates were further validated by qRT-PCR and showed high predictive power, sensitivity and specificity in a replication cohort. Later, miR-331-5p was shown as the only significantly increased miRNA in plasma of PD patients using qRT-PCR in a cohort with similar numbers (Cardo et al., 2013). Li et al. assessed the levels of 3 miRNAs, which were previously reported to associate with neurogenesis and PD-related processes (Cheng et al., 2009; Gehrke et al., 2010; Kong et al., 2015). They found two significantly regulated miRNAs, miR-137-3p, and miR-124-3p, in PD patients (Li et al., 2017). In a similar study the expression of 5 pre-selected miRNAs was analyzed, identifying increased expression of miR-30a-5p and miR-30b-5p in PD (Schwienbacher et al., 2017). Five studies used serum to find potential miRNA biomarkers in PD. In 2014, serum levels of several miRNAs in a discovery cohort were investigated (10 idiopathic/10 LRRK2-mutated/ 10 controls) by qRT-PCR. The findings were validated in two isolated replication studies (in total 85 idiopathic PD patients, 11 LRRK2-mutation carriers and 85 controls), where the expression levels of four miRNAs (miR-29a-3p, miR-29c-3p, miR-19a-3p, and miR-19b-3p) were significantly reduced in idiopathic PD patients and LRRK2-mutation carriers (Botta-Orfila et al., 2014). A study including a significantly larger number of subjects in both discovery and validation cohorts distinguished a set of 5 serum-miRNAs that are able to differentiate PD patients from controls: after Solexa-sequencing and PCR validation miR-195-5p, miR-185-5p, miR-15b-5p, miR-221-3p, and miR-181a-5p were differentially expressed in PD (Ding et al., 2016). Vallelunga and colleagues assessed miRNA levels in the serum of PD patients, multiple systems atrophy (MSA) and control subjects, revealing miRNAs specifically downregulated in PD (miR-30c-5p and miR-148b-3p) (Vallelunga et al., 2014). Similarly, another study found down-regulation of miR-141-3p, miR-214-3p, miR-146b-5p, and miR-193a-3p in PD. Individual receiver-operating-characteristic (ROC) curves for the miRNAs presented high area under the curve (AUC) values for both training and validation cohorts (AUCs > 0.782) (Dong et al., 2016). Evaluation of miRNAs in serum of 138 PD patients and 112 controls by qRT-PCR identified 4 candidate miRNAs in PD (miR-29c-3p, miR-146a-5p, miR-214-3p, and miR-221-3p). Among those, miR-221 was found decreased and showed a positive correlation to UPDRS scores and an AUC value of 0.787 for PD-prediction (Ma et al., 2016). Again, all mentioned studies show a very limited overlap in detected target miRNAs and only miR-29c-3p, miR-214-3p, and miR-221-3p were reported in more than one study. Compared to the studies in plasma, the cohorts here were much larger, which might explain their better reproducibility.

miRNA expression in PBMCs of PD patients was addressed in 4 studies. In 2011 microarray analysis of 19 PD and 13 control cases identified 18 miRNAs as down-regulated in PD, including several of the regulated miRNAs in serum/plasma mentioned above (Martins et al., 2011). Furthermore, it was shown that deep brain stimulation (DBS) influences miRNA expression in PD patients. 16 differentially expressed miRNAs were found in leukocytes of PD patients in comparison to controls pre-DBS. Post-DBS 11 miRNAs were significantly regulated in relation to the pre-DBS state. Interestingly, 5 miRNAs were common between both comparisons but inversely regulated post-DBS, suggesting that this therapeutic intervention has an influence on miRNA expression in PBMCs (Soreq et al., 2013). Another study revealed up-regulation of miR-29c-3p, miR-424-5p, and miR-30e-5p in PBMCs of PD cases, partially in line with the aforementioned findings (Pasinetti, 2012). At last, qRT-PCR analysis of 5 pre-selected miRNAs revealed increased expression of miR-103a-3p, miR-30b-5p, and miR-29a-3p in PBMCs of PD patients (Serafin et al., 2015). All mentioned studies were performed with relatively small cohorts (7–46 PD patients) and show no overlapping results. Interestingly, the studies by Martins et al. and Serafin et al. both report differential expression of miR-30b-5p in PD, however, with inversed directions.

One of the few studies investigating miRNA levels in CSF identified differentially expressed miRNAs in post-mortem serum and CSF samples of PD patients and controls using NGS. Besides reporting that the miRNA signature in CSF seems to be slightly more stable, they found 17 differentially expressed miRNAs in CSF (Burgos et al., 2014). The authors observed a decrease in miR-132-5p PD, which is an extensively studied miRNA linked to PD (Gillardon et al., 2008; Junn and Mouradian, 2012; Yang et al., 2012). In the serum of PD patients 5 miRNAs were found deregulated, which partially overlapped with other studies (Margis et al., 2011; Schwienbacher et al., 2017).

Quantitative RT-PCR analysis of 44 PD and 42 controls identified miR-200a-3p, miR-542-3p, and miR-144-5p upregulated in CSF of PD patients. After ROC and ordinal regression analyses, these candidates showed a high correlation with disease stages (Mo et al., 2016). In a similar study analyzing CSF of PD and MSA cases, levels of miR-205 and miR-24 were found decreased in PD (Marques et al., 2017).

**FIGURE 2:**
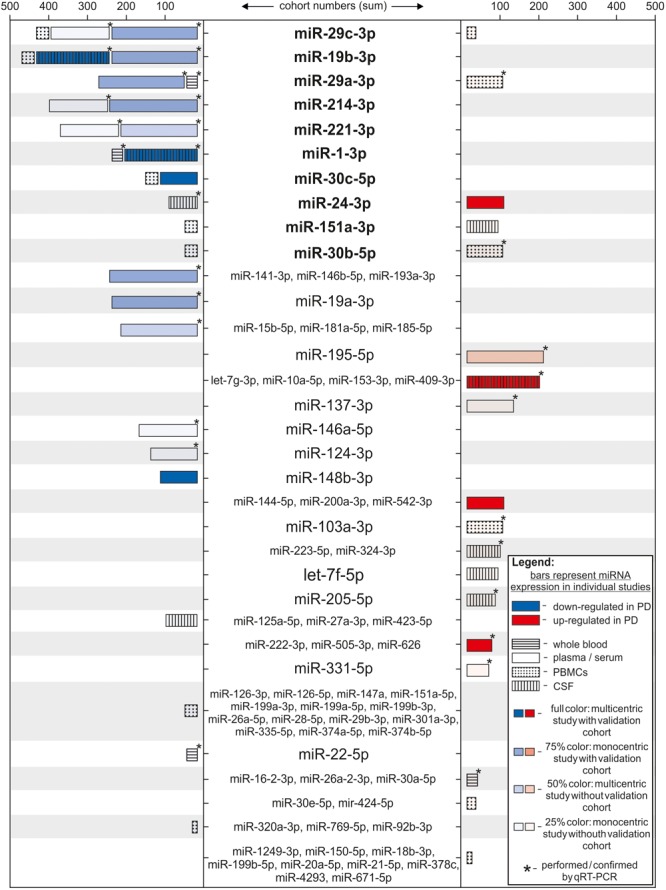
Differentially regulated miRNAs ranked according to strength of the study. Each bar represents one individual study, where the miRNA(s) indicated in the middle column was identified differentially regulated. miRNA names are set bold when appearing in more than one study. Length of the bars correspond to the number of subjects in the study cohort (patients + controls). Color of the bars represents significant down- (blue) or up-regulation (red) in miRNA expression in respect to the PD group. Color intensity indicates strength of the study by considering the number of centers for sample recruitment and existence of a validation cohort per study (full color: multicentric study with validation cohort; 75% color: monocentric study with validation cohort; 50% color: multicentric study without validation cohort; 25% color: monocentric study without validation cohort). Bars are patterned according to the biological source used in each study (horizontally striped: whole blood; solid: plasma or serum; dotted: PBMCs; vertically striped: CSF). ^∗^ = studies where results were acquired or confirmed using qRT-PCR.

Exosomal miRNAs isolated from CSF also show differential expression according to disease states. 27 significantly regulated miRNAs were identified in CSF exosomes by qRT-PCR (Gui et al., 2015). Thereof, 6 miRNAs were further validated based on their involvement in related signaling pathways: miR-1, miR-19b-3p showed decreased expression in PD, while miR-153, miR-409-3p, miR-10a-5p, and let-7g-3p were found upregulated. A recent study reported that 5 miRNAs were able to differentiate between early stage PD and healthy controls: miR-151a-3p and let-7f-5p were up-regulated in PD CSF, whereas miR-27a-3p, miR-125a-5p, and miR-423-5p were significantly decreased (dos Santos et al., 2018). The different studies performed with total CSF or CSF-exosomes show no overlapping results, even though the cohorts were of a comparable size. One possible explanation are the different methods used for analysis and the fact that RNA was isolated from total CSF or CSF-exosomes. Furthermore, Burgos and colleagues used CSF that was taken post-mortem. Taken together, several miRNAs have been reported as differentially expressed in multiple body fluids of PD patients (**Supplementary Table 1**). Only a few of them were significantly regulated in more than one study, e.g., miR-29c-3p, miR-19b-3p, and miR-29a-3p, partially with contradictory results that might be explained by differences in the technical execution (**Figure 2**). Reasons for the limited overlap between studies can be found on different levels of the analyses. On one hand, the studies mentioned above use different source material for their analysis, mainly serum, plasma, PBMCs, or CSF, leading to different results. Direct comparisons of blood- and CSF-derived miRNAs revealed different miRNA expression patterns as well as blood- or CSF-exclusive miRNAs that were not detected in the respective other body fluid (Sørensen et al., 2014, 2016). This implements that not-blood-derived source materials like CSF have to be controlled extensively for blood contamination (Müller et al., 2014). In addition, cohort size differed between the studies and not all included an independent validation cohort. Another factor that might lead to different results is the method of RNA isolation. Depending on the isolation method (e.g., total RNA vs. small RNA; guanidinium thiocyanate-phenol-chloroform extraction vs. lysis followed by column precipitation; choice of RNA carrier) used, different size profiles and RNA quantities can be expected. This was demonstrated in studies analyzing the influence of the RNA isolation method on miRNA expression (Wang et al., 2008; Eldh et al., 2012; Burgos et al., 2013; McAlexander et al., 2013). Finally, the method to detect and subsequently analyze miRNA expression levels strongly influences the outcome of the studies. As NGS results in the complete quantitative and qualitative miRNAome of a sample, it provides more information than microarrays and qRT-PCR where targets are pre-selected. The studies included in this review show differences in several aforementioned aspects and thus their comparability is limited. However, **Figure 2** shows that some miRNA targets are reproducible, in some cases even in different biomarker sources (miR-19b-3p and miR-1a-3p). Interestingly, contradictory results are only reported for two miRNAs within the same biomarker source (miR-29c-3p, miR-30b-5p in PBMCs) (Martins et al., 2011; Pasinetti, 2012; Serafin et al., 2015).

Most studies mentioned here performed ROC curve analysis to estimate the diagnostic accuracy of the proposed miRNAs. The diagnostic accuracy for single miRNAs ranged between 63% (Ma et al., 2016) and 97% (Gui et al., 2015) and was improved when multiple miRNAs were combined to a panel (77% (Botta-Orfila et al., 2014) to 99% (Gui et al., 2015)). Compared to the average diagnostic accuracy based on clinical criteria, which varies between 73.8% (examination by non-experts) and 83.9% (examination by movement disorder experts after follow-up) (Rizzo et al., 2016), some of the proposed miRNA biomarkers have a higher diagnostic value and discriminated PD cases better from controls than the clinical assessment alone. This, however, suggests that adding a miRNA biomarker (panel) during diagnosis might improve the diagnostic accuracy for idiopathic PD. An attempt to functionally annotate targets of the top 3 miRNAs (miR-29c-3p, miR-19b-3p, and miR-29a-3p) reveals a plethora of pathways that are proposed to be regulated. Among those, we find multiple intracellular signaling cascades, but also disease-related pathways. Contrary to what one would potentially assume, an emerging unifying theme is yet missing. This may reflect the heterogeneity of the disease as well as the difference in source fluids and miRNA detection methods. Further studies with larger patient numbers and a higher methodical standardization might sharpen the picture here.

## Circulating miRNAs as Biomarkers in Other Parkinsonian Syndromes

Although clinically, the differential diagnosis of PD versus other Parkinsonian syndromes is more challenging than the diagnosis of PD alone, only a few studies are available concerning the diagnostic value of miRNAs in this field. The best evidence so far is available for MSA.

Analyses of miRNAs in plasma of 34 MSA patients, 31 PD patients and 30 controls by microarrays revealed 12 miRNAs differentially regulated in MSA, of which five were validated by qRT-PCR (miR-24, miR-148b, miR-223, miR-324-3p, and miR-339-5p) (Vallelunga et al., 2014). Comparing MSA and PD, of 5 differentially regulated miRNAs in the microarrays, only 3 showed significantly increased expression after qRT-PCR validation (miR-24, miR-34b, and miR-148b). Target analysis showed an involvement of those miRNAs in cell cycle regulation, apoptosis, and post-translational modifications. Kume and colleagues investigated serum of 10 MSA patients and 6 controls. Microarray analysis showed the up-regulation of 50 miRNAs in MSA patients, whereas 17 miRNAs were significantly decreased (Kume et al., 2017). Again, miR-223, and miR-24 were found to be up-regulated, but qRT-PCR validation of the most regulated miRNAs (miR-223 and miR-16) showed negative results. Another group defined a set of plasma miRNAs using 32 PD and 32 controls and a validation cohort (42 PD and 30 controls) (Khoo et al., 2012). Here, miR-626, and miR-505 showed some value in the classification of 4 MSA and 5 Progressive Supranuclear Palsy (PSP) patients, which were part of an additional validation cohort.

Contrary to the aforementioned increase in miR-24 expression in serum of MSA patients, analyses of 10 preselected miRNAs in CSF of MSA, PD and control patients revealed a decreased miR-24 expression in MSA and PD (Marques et al., 2017). The regulation of miR-148b in MSA, which was described in blood samples, could not be reproduced. Furthermore, miR-19a, miR-19b, and miR-34c were also found to be decreased in MSA. PARK2, LRRK2, and VPS35 are predicted targets for miR-19a and miR-19b, while SNCA, PLA2G6, and SLC1A4 are targets for miR-34c. Taken together, the studies investigating miRNA biomarkers for MSA show a limited overlap of single deregulated miRNAs (e.g., miR-223 and miR-24 in blood). However, there appear to be marked differences between miRNA expression in blood and CSF as miR-24 show an inverse regulation in these source fluids. As in all studies the cohorts are very small and different materials and methods were used, the comparability is limited and more studies with standardized procedures are needed to establish a miRNA biomarker with good diagnostic accuracy. Interestingly, in patients with idiopathic rapid eye movement sleep behavior disorder, a condition that is considered as an early pre-motor stage of synucleinopathies, downregulation of miR-19b in serum samples might predict the conversion into PD or Dementia with Lewy Body (DLB) and mimics the situation in the CSF of MSA patients (Fernández-Santiago et al., 2015). A multicenter study primarily focusing on Alzheimer’s disease also included CSF samples of DLB patients. Analyzing 6 different miRNAs, miR-125b was found decreased in DLB compared to the non-demented controls, but only before correcting for covariates (Müller et al., 2016). Idiopathic normal pressure hydrocephalus (iNPH) is a frequent clinical PD mimic. A recent analysis analyzed the CSF of 81 iNPH patients together with samples from 28 patients with a possible Parkinsonian syndrome (iNPH in combination with an abnormal metaiodobenzylguanidine or 123I-Ioflupane dopamine transporter scan (DaTSCAN)) (Jurjević et al., 2017). Here, miR-4274 was down-regulated compared to patients without evidence of dopaminergic denervation.

## Concluding Remarks

The analysis of miRNA as biomarker for neurodegenerative disorders is a rapidly evolving field, which entered the stage not even a decade ago. Already, numerous studies have revealed a number of circulating miRNAs as potential biomarkers for PD and other Parkinsonian syndromes. Although the overlap between the candidates is low in different studies, this might be a result of differences in several methodological and technical aspects, ranging from the biomarker source material to RNA quality and isolation methods to detection technologies and finally statistical evaluations applied to the acquired data. However, the findings gathered here evidence that miRNAs can be readily detected and found differentially expressed in multiple body fluids and thus could represent promising diagnostic biomarkers for PD and other Parkinsonian syndromes. In order to further develop a diagnostic biomarker for clinical use, stringent standardization of all methodological aspects as well as study and validation cohorts from multiple centers with higher patient numbers and preferentially post-mortal verification of the diagnosis increasing the diagnostic accuracy have to be ensured. Additionally, fast, reliable, easy-to-handle, and cost-efficient miRNA detection methods are needed. Future studies will also have to determine the value of miRNAs as prognostic markers and answer the question whether miRNAs correlate with disease progression in PD. This would yield highly valuable information for clinical trials with disease-modifying therapies.

## Author Contributions

AR and LCG developed the idea under the lead of PL, performed literature research, wrote and finalized the manuscript, and prepared the figures. FM and JS were involved in literature research and writing of the manuscript. PL developed the idea for this review and revised the manuscript. All authors have seen and approved the final version.

## Conflict of Interest Statement

The authors declare that the research was conducted in the absence of any commercial or financial relationships that could be construed as a potential conflict of interest.
