# Supplementary material for: Circulating miRNAs as Diagnostic Biomarkers for Parkinson’s Disease
Source: Front Neurosci. 2018 Sep 5;12:625. doi: 10.3389/fnins.2018.00625 (PMC6135037; doi:10.3389/fnins.2018.00625)
Supplement: Supplementary file 1 [file Data_Sheet_1.PDF]

Supplementary Material

Circulating miRNAs as diagnostic biomarkers for Parkinson's disease

Anna-Elisa Roser#, Lucas Caldi Gomes#, Jonas Schünemann, Fabian Maass, Paul Lingor*

*** Correspondence:** Paul Lingor: plingor@gwdg.de

Supplementary Table 1: Studies using circulating miRNAs as biomarkers for PD or other Parkinsonian syndromes.

Author, year	source	method	study type (n° of centres)	deregulated miRNAs in PD	deregulated miRNAs in other Parkinsonian syndromes	Discovery cohort	validation cohort	reported relevant pathways
Margis et al., 2011	whole blood	microarrays/ qRT-PCR	Mono-centric	↑ miR-16-2-3p, miR-26a-2-3p and miR-30a-5p (in PD treated vs PD untreated);<br>↓ miR-1-3p, miR-22-5p, miR-29a-3p (PD vs Ctr)	n/a	8 non-treated PD,<br>4 treated PD,<br>7 early onset PD,<br>8 CTRL	-	- tubulin/microtubule system and αSyn aggregation<br>- dopamine transport regulation<br>- protein kinase C-mediated internalization of the dopamine transporter (DAT)<br>- glutamatergic regulation<br>- BDNF signalling
Khoo et al., 2012	plasma	microarrays/ qRT-PCR	Multi-centric (2)	↑ miR-1826, miR-222-3p, miR-626 and miR-505-3p	↑ miR-626, miR-505-3p	32 PD,<br>32 CTRL	30 PD,<br>8 CTRL,<br>5 PSP,<br>4 MSA (val. cohort);<br>42 treated PD,<br>30 CTRL (replication cohort)	- cell proliferation and survival<br>- multiple sclerosis (MS)
Cardo et al., 2013	plasma	qRT-PCR	Mono-centric	↑ miR-331-5p	n/a	31 PD, 25 CTRL	-	- neurogenesis and neurodegeneration<br>- axon guidance<br>- MAPK signalling
Li et al., 2017	plasma	qRT-PCR	Mono-centric	↑ miR-137-3p;<br>↓ miR-124-3p	n/a	60 PD,<br>60 CTRL	-	- Parkin-induced mitochondrial autophagy<br>- calpain/cdk5 pathway proteins in dopaminergic neurons<br>- loss of dopaminergic neurons in MPTP-treatment<br>- apoptosis and impaired autophagy of SNpc dopaminergic neurons<br>- proliferation and differentiation of adult neural stem cells<br>- depression

Schwienbacher et al., 2017	plasma	qRT-PCR	Mono-centric	↑ miR-30a-5p and miR-30b-5p *(trend)*	n/a	50 treated PD, 10 non-treated PD, 49 CTRL	-	- EGFR/PI3K/Akt pathway<br>- PI3K/Akt/mTOR pathway<br>- oxidative stress / increased susceptibility to neurotoxicity<br>- mitochondrial dynamics
Botta-Orfila et al., 2014	serum	qRT-PCR	Mono-centric	↓ miR-29a-3p, miR-29c-3p, miR-19a-3p and miR-19b-3p	n/a	10 PD, 10 LRRK2 PD, 10 CTRL	20 PD, 20 LRRK2 PD, 20 CTRL (val. cohort 1); 65 PD, 65 CTRL (val. cohort 2)	- extracellular matrix reception (ECM) pathway / leucocyte recruitment<br>- adipocytokines<br>- focal adhesion<br>- axon guidance<br>- mTOR sig. pathway<br>- MAPK sig. pathway
Vallelunga et al., 2014	serum	microarrays	Multi-centric (2)	↑ miR-24-3p, miR-324-3p and miR-223-5p; ↓ miR-30c-5p and miR-148b-3p	↑ miR-24-3p, miR-148b-3p, miR-223-3p, miR-324-3p; ↓ miR-339-5p	6 PD, 6 MSA-P, 3 MSA-C, 5 CTRL	25 PD, 25 MSA, 25 CTRL (val. cohort)	- cell cycle regulation<br>- modulation of apoptosis<br>- spinal cord injury<br>- response to neuronal injury<br>- demyelination in multiple sclerosis
Fernández-Santiago et al., 2015	serum	qRT-PCR	Mono-centric	n/a	↓ miR-19b-3p, miR-29a-3p and miR-29c-3p	RBD1 (disease-free), RBD2 (PD or DLB), 28 CTRL	-	- regulation of PD-associated genes
Ding et al., 2016	serum	Solexa-sequencing / qRT-PCR	Multi-centric (2)	↑ miR-195-5p; ↓ miR-185-5p, miR-15b-5p, miR-221-3p and miR-181a-5p	n/a	106 PD, 91 CTRL	-	- astrocyte dysfunction<br>- dendritic and spine development deficits<br>- neuron apoptosis, development, regeneration and growth<br>- Alzheimer’s disease / MS
Dong et al., 2016	serum	Solexa-sequencing / qRT-PCR	Mono-centric	↓ miR-141-3p, miR-214-3p, miR-146b-5p and miR-193a-3p	n/a	30 PD, 30 CTRL	92 PD, 74 CTRL	- regulation of PD-associated genes<br>- selective dopaminergic neural cell death and absence of Lewy bodies by PARK2 regulation<br>- neuron guidance, growth, polarity/axonal growth, neural

								differentiation, proliferation and apoptosis by PDDC1 and DGKQ regulation<br>- neuroinflammation<br>- fibrosis
Ma et al., 2016	serum	qRT-PCR	Mono-centric	↓ miR-29c-3p, miR-146a-5p, miR-214-3p and miR-221-3p	n/a	138 PD, 112 CTRL	-	- extracellular matrix (ECM)-receptor interaction<br>- focal adhesion<br>MAPK, Wnt and mTOR signalling<br>- neuronal projection<br>- regulation of αSyn<br>- neuroinflammation initiation<br>- iron accumulation<br>- apoptosis / fibrosis
Kume et al., 2017	serum	microarrays	Mono-centric	n/a	↑ 50 miRNAs; ↓ 17 miRNAs *no significance for selected species by qRT-PCR validation*	10 MSA, 6 CTRL	-	- αSyn aggregation / neuronal apoptosis by HSP70 regulation<br>- regulation of immune response<br>- autophagy<br>- inflammation<br>- cell proliferation and apoptosis by IGF-1 regulation<br>- neurodegeneration<br>- Alzheimer's disease
Martins et al., 2011	PBMCs	microarrays	Mono-centric	↓ miR-335-5p, miR-374a-5p, miR-199a-3p, miR-199b-3p, miR-126-5p, miR-151a-3p, miR-199a-5p, miR-151a-5p, miR-126-3p, miR-29b-3p, miR-147a, miR-28-5p, miR-30b-5p, miR-374b-5p, miR-19b-3p, miR-30c-5p, miR-29c-3p, miR-301a-3p and miR-26a-5p	n/a	19 PD, 13 CTRL	-	- protein ubiquination pathway - DNA methylation and transcriptional repression signalling<br>- semaphorin signalling in neurons<br>- retinoic acid receptor activation<br>- glycosphingolipid biosynthesis<br>- synaptic long-term potentiation
Pasinetti, 2012	PBMCs	NGS	Mono-centric	↑ miR-29c-3p, miR-424-5p and miR-30e-5p	n/a	13 PD, 10 CTRL	-	- PD pathophysiology / cellular dopamine metabolism and release

Soreq et al., 2013	PBMCs	NGS	Mono-centric	↑ miR-21-5p, miR-671-5p, miR-150-5p, miR-1274b and miR-199b-5p; ↓ miR-320a-3p, miR-92b-3p, miR-769-5p, miR-320b-1/miR-320b-2, miR-320c-1/miR-320c-2, miR-16-1/miR-16-2 (precursors), ↑miR-1249-3p, miR-20a-5p, miR-18b-3p, miR-378c, miR-4293 *(the last 5 on the list had inverted expression after DBS)*	n/a	7 PD, 6 CTRL	-	- mitochondrion organization and metabolism<br>- ubiquitin homeostasis / ligase complex<br>- leukocyte/disease related pathways<br>- regulation of translation<br>- MAPK phosphatase export from nucleus<br>- neuroinflammation<br>- regulation of NK cell cytotoxicity<br>- regulation of amyloid precursor protein<br>- neurodegenerative disorders including prion-induced neurodegeneration, PD, AD and Huntington’s disease
Serafin et al., 2015	PBMCs	qRT-PCR	Mono-centric	↑ miR-103a-3p, miR-30b-5p, and miR-29a-3p	n/a	36 treated PD, 10 non-treated PD, 46 CTRL	-	- neurodegenerative disorders including PD<br>- AKT/PTEN pathway regulation<br>- dopaminergic neuron development by regulation of BCL2<br>- cell death and oxidative stress by DJ-1 regulation<br>- neurotoxicity related to GPR37 regulation<br>- neuronal death related to CDC42 regulation
Burgos et al., 2014	CSF/ serum *post-mortem*	NGS	Mono-centric	↑ miR-19a-3p, miR-19b-3p and let-7g-3p; ↓ miR-132-5p, miR-485-5p, miR-127-3p, miR-128, miR-409-3p, miR-433-3p, miR-370, miR-431-3p, miR-873-3p, miR-136-3p, miR-212-3p,	n/a	67 PD patients, 78 CTRL	-	- regulation of αSyn levels / accumulation<br>- regulation of amyloid precursor protein<br>- memory consolidation<br>- synaptic plasticity by SIRT1 regulation<br>- cognitive decline

				miR-10a-5p, miR-1224-5p, miR-4448 *in CSF*; ↑ miR-338-3p, miR-30e-3p and miR-30a-3p; ↓ miR-16-2-3p, miR-1294-5p, *in serum*				- regulation of mitochondrial function<br>- neuronal cell death, oxidative stress and αSyn aggregation by DJ-1 regulation<br>- neuroinflammation<br>- dopaminergic cell development / differentiation<br>- neurodegenerative diseases including PD and AD
Gui et al., 2015	CSF	qRT-PCR	Multi-centric (2)	↑ miR-153-3p, miR-409-3p, miR-10a-5p and let-7g-3p;<br>↓ miR-1-3p, miR-19b-3p,	n/a	47 PD,<br>27 CTRL	78 PD,<br>35 CTRL	- neurotrophin signaling pathway<br>- mTOR signaling pathway<br>- ubiquitin mediated proteolysis<br>- long-term potentiation<br>- Axon guidance<br>- cholinergic, glutamatergic and dopaminergic synapsis
Mo et al., 2016	CSF	qRT-PCR	Mono-centric	↑ miR-200a-3p, miR-542-3p and miR-144-5p	n/a	44 PD,<br>42 CTRL	-	- regulation of neuronal differentiation and proliferation<br>- ischemic stroke and intracerebral hemorrhage<br>- Huntington's and Alzheimer's disease
Müller et al., 2016	CSF	qRT-PCR	Mult-centric (3)	n/a	↓ miR-125b-5p	37 DLB,<br>40 CTRL	-	n/a
Marques et al., 2017	CSF	qRT-PCR	Mono-centric	↑ miR-205-5p; ↓ miR-24-3p	↓ miR-24-3p, miR-19a-3p, miR-19b-3p, miR-34c-5p	28 PD,<br>17 MSA,<br>28 CTRL	-	- involvement with idiopathic rapid eye movement sleep behavior disorder<br>- regulation of Parkinson's disease related genes including LRRK2, DJ1, parkin and αSyn
Jurjević et al., 2017	CSF	qRT-PCR	Multi-centric (2)	n/a	↓ miR-4274 in the PS group	81 iNPH (28 with a possible	-	- involvement in dopaminergic signalling pathway: regulation of active transport of dopamine into

						Parkinsonian syndrome – PS), 6 CTRL		synaptic vesicles and other types of secretory vesicles for dopamine exocytotic release (by SLC18A2 regulation)
dos Santos et al., 2018	CSF	NGS	Mono-centric	↑ miR-151a-3p and let-7f-5p; ↓ miR-27a-3p, miR-125a-5p and miR-423-5p	n/a	40 PD, 40 CTRL	-	- prion disease<br>- TGF-beta signalling<br>- cell cycle regulation<br>-ubiquitin-mediated proteolysis<br>- neurotrophin signalling<br>- mTOR signalling<br>- AMPK signalling<br>- FoxO signalling<br>- fatty acid biosynthesis<br>- Huntington’s disease
